# Development of a Photostable pH Biosensor Based on mStayGold

**DOI:** 10.1002/cbic.70512

**Published:** 2026-08-26

**Authors:** Michael Chang, Lilly Nash, Ruben Bierings, Kei Takahashi‐Yamashiro, Takuya Terai, Tom Carter, Robert E. Campbell, Kelvin K. Tsao

**Affiliations:** ^1^ Department of Chemistry Graduate School of Science The University of Tokyo Bunkyo‐ku Tokyo Japan; ^2^ Department of Biomedical Sciences The Royal Veterinary College London UK; ^3^ Department of Molecular & Biomedical Sciences St. George’s School of Health and ­­Medical Sciences City St. George’s University of London London UK; ^4^ Department of Hematology Erasmus University Medical Center Rotterdam Netherlands; ^5^ Core Research for Evolutional Science and Technology (CREST) Japan Science and Technology Agency (JST) Chiyoda‐ku Tokyo Japan; ^6^ CERVO Brain Research Center and Department of Biochemistry Microbiology and Bioinformatics Université Laval Québec Canada; ^7^ Global Standard Science Education Graduate School of Science The University of Tokyo Bunkyo‐ku Tokyo Japan

**Keywords:** biosensor, carbon dioxide, directed evolution, exocytosis, fluorescent protein, pH, protein engineering, Weibel‐Palade bodies

## Abstract

Fluorescent proteins (FPs) that are pH‐sensitive play a crucial role in investigating pH‐related cellular processes, such as endocytosis and exocytosis. Existing pH‐sensitive FPs generated from *Aequorea victoria* green fluorescent protein (GFP), such as superecliptic pHluorin (SEP) and Lime, have been widely employed to study these processes, but suffer from low photostability. Here, we report the development and characteristics of serapH, a genetically encodable pH biosensor with improved photostability compared to GFP analogues, which we generated using mStayGold as a scaffold. To aid in the development of serapH, we developed a method for screening pH‐sensitive FP variants by directly evaluating both brightness and pH sensitivity in bacterial colonies on agar. This significantly increased the number of colonies that could be screened per round and reduced the time needed per round. The photostability of serapH should improve spatiotemporal resolution by increasing tolerance to higher excitation intensities and longer imaging durations, thereby expanding the range of applications of pH‐sensitive FPs.

## Graphical abstract

The front cover shows the iterative directed evolution process used to evolve the biosensor. Incubation with CO_2_ allowed for direct screening of both fluorescence and pH sensitivity in mutant colonies on agar. Our efforts produced serapH1.0, a photostable pH biosensor that is highly fluorescent at physiologically neutral pH (7.4) and dim at acidic pH (5.5).

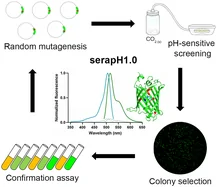

## 1 Introduction

Cellular pH is a fundamental component of many biological processes. The acidic secretory organelles of the cell are one such example: synaptic vesicles have a pH of approximately 5.5 in their lumen [1, 2], while the Weibel‐Palade bodies (WPBs) of endothelial cells, which store the helical tubules of Von Willebrand factor (VWF) [3, 4], have a resting pH ranging from 5 to 5.5. When these structures undergo membrane fusion during exocytosis, exposure to the extracellular environment causes a shift to pH 7.0–7.4 for the vesicular contents. Likewise, cellular structures meant for storage or degradation, such as lysosomes and endosomes, have a substantially lower pH than their surrounding intracellular environment, which in turn influences the behavior of proteins and molecules within them. Researchers can obtain valuable insights into the nature of these processes by visualizing the local changes in pH that accompany them. To this end, genetically encoded fluorescent proteins (FPs) that are particularly sensitive to pH changes in the physiologically relevant range are important tools for cellular research [5, 6, 7]. These minimally disruptive tools have enabled the visualization of synaptic events [2, 8] and neurotransmission [9, 10] in real time, highlighted the trafficking of membrane proteins to acidic locales [11, 12, 13], and have been used to study the mechanisms and signaling pathways underlying exocytosis [14, 15, 16]. Using the *Aequorea victoria* green fluorescent protein (GFP) as a scaffold, current FP‐based pH sensors such as Lime have achieved impressive levels of performance, with high brightness and a fluorescent response up to 80‐fold between pH 5.5 and 7.4 [17] (Table S1). One major limitation, however, is their low photostability, which is a significant issue since vesicular events occur rapidly and high frame rate imaging is required. This is typically addressed by using total internal reflection fluorescence (TIRF) imaging, which selectively excites fluorophores near the plasma membrane, increasing image contrast and reducing photobleaching in the bulk of the cell [18]. However, TIRF limits imaging to a thin area near the plasma membrane, making it difficult to monitor protein trafficking to intracellular structures across the whole cell [19].

**FIGURE 1:**
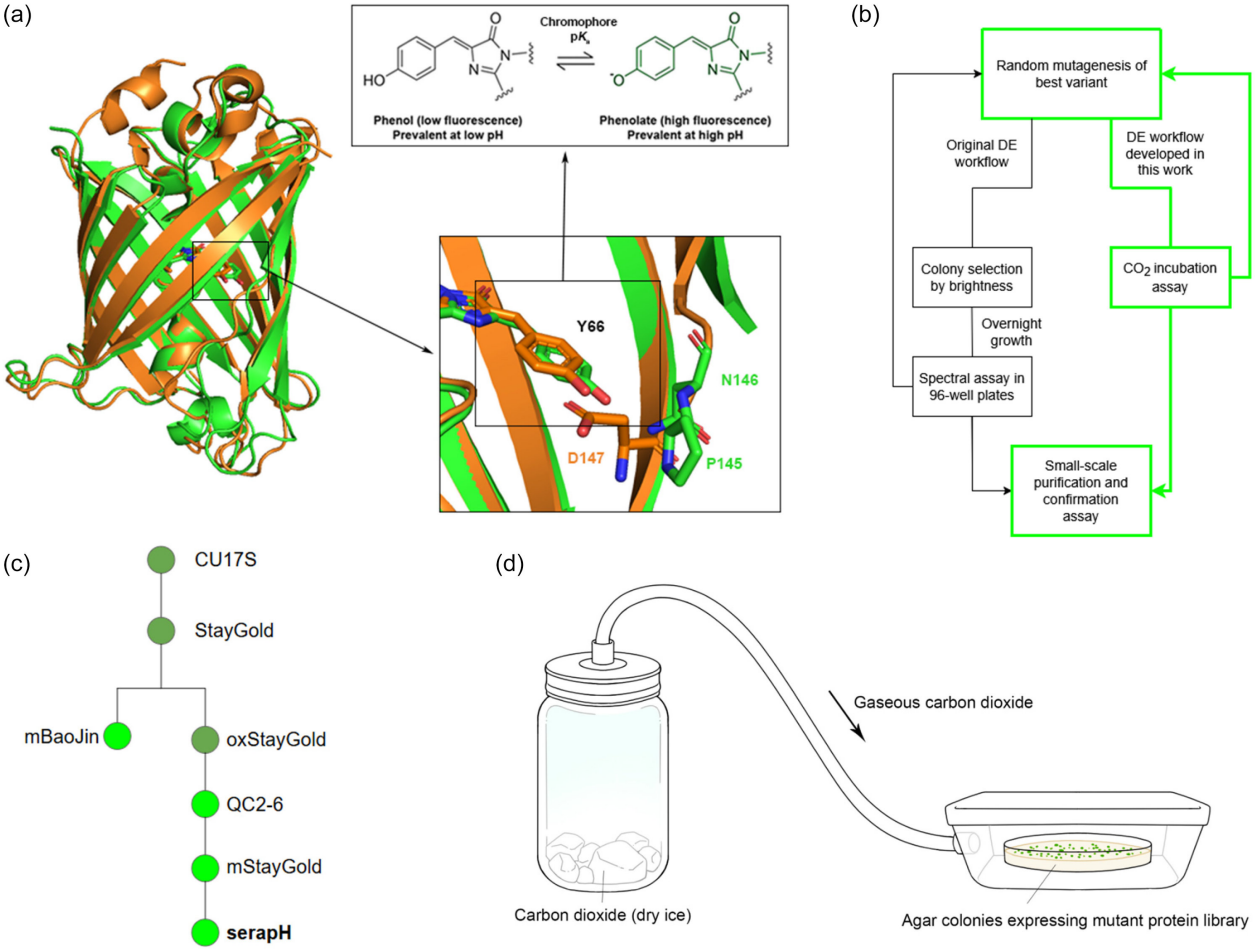
Premise of pH sensor development from mStayGold. (a) Overlaid structures of StayGold (green, PDB ID: 8BXT) and pH sensor Lime (orange, PDB ID: 7YV3). Phenol‐phenolate equilibrium of the chromophore Y66 residue dictates the brightness of FPs at different pH levels. The p*K*_a_‐altering interaction between Y66 and D147 in Lime formed the rational basis for the creation of a pH sensor from mStayGold. (b) Accelerated evolutionary workflow using direct screening on agar plates, showing the standard approach (left) and using the CO_2_ assay presented in this work (right, green). (c) Protein lineage of serapH development from StayGold. (d) Representative diagram of assay chamber for CO_2_‐based screening of pH‐sensitive FPs. Incubation with CO_2_ leads to a transient decrease in pH within the agar colonies.

Most FPs display some intrinsic pH sensitivity due to the equilibrium between the protonated (dim, with an absorbance maximum ~400 nm) and deprotonated (bright, with an absorbance maximum ~480 nm) states of the chromophore (Figure 1a). However, their p*K*_a_ is typically too low to reliably reflect changes in physiological pH, that is, between 5.5 and 7.4 [2, 8] (Table S1). To develop and tune pH sensitivity in a given FP, two potential strategies can be used: structure‐guided rational mutation of key residues and directed evolution. Many efforts to develop pH‐sensitive FPs employ a mix of both methods, with rational targeting used to create an initial prototype and directed evolution to improve the sensor’s properties beyond that. This sequential approach was used to create the first genetically encoded GFP‐based pH biosensor, super‐ecliptic pHluorin (SEP) [8, 20]. The same approach has been applied to the development of other GFP‐based pH sensors, including EGFP [21] and Lime [17], in which rational mutagenesis of residues surrounding the chromophore is paired with library screening to tune protein p*K*_a_ into the physiological range. However, in each case, library screening involved the selection of variants from the same colony‐picking and multiwell assay workflow. Directed evolution often constitutes the most time‐ and labor‐intensive step of development. One of the most established and common methods of evolution involves generating mutant plasmid libraries and evaluating them through colony‐based screening of *Escherichia coli*. Since the selection of optimal variants depends on both brightness and analyte response, colonies typically need to be screened in a two‐step system: an initial selection for brightness on agar plates to identify correctly folded proteins, followed by a secondary screening where the proteins extracted from specific colonies are assayed for their response, typically in multiwell plate format [22] (Figure 1b, left). This severely constrains both the throughput and speed of each evolutionary round. To overcome this limitation, researchers have employed a variety of automation‐based screening platforms, such as fluorescence‐activated cell sorting (FACS) [23, 24], or direct screening of fluorescent response for analyte‐dependent biosensors in rat neurons and mammalian cells using microscopy and image analysis [25, 26, 27]. However, cost and training barriers limit the accessibility of these methods, particularly in academic research settings. On top of this, mammalian cell platforms generally have limited throughput and high specificity for their targeted analyte, with no platform to date developed for pH biosensor development.

Here, we report the development of a new pH biosensor based on monomeric StayGold. Unlike most other green FPs, which originate from GFP first isolated from *A. victoria*, StayGold was derived from the FP CU17S originating in *Cytaeis uchidae*, which demonstrated high photostability [28, 29] (Figure 1c). A single point mutation increased the brightness of the protein, creating StayGold, and two separate efforts have yielded the monomeric variants mStayGold(J) [29] and mBaoJin [30]. We selected mStayGold(J) (hereafter called mStayGold or mSG) as a scaffold due to its exceptionally high photostability and suitability for protein tagging. After performing rational site‐directed mutagenesis to create an initial prototype, we used directed evolution to generate a pH biosensor with a p*K*_a_ of about 7.1 and approximately 21‐fold intensiometric response from pH 5.5 to pH 7.4. This dynamic range is well matched to the physiological window where pH changes occur while retaining the photostability of mSG. This sensor, which we named serapH to reflect its strengths of longevity and luminance, demonstrates characteristics well‐suited for live monitoring of physiological pH, including photostability similar to that of mStayGold. Finally, we demonstrate the performance of serapH in a physiological setting by successfully imaging exocytosis of WPBs.

## 2 Results

### 2.1 Development of an mStayGold‐Based pH Sensor Through Directed Evolution

Before performing evolution, we generated an initial serapH prototype through site‐saturation mutagenesis (SSM) of two residues in the β‐barrel near the chromophore using the 22‐Codon technique (22C). 22C has been shown to produce fewer redundant mutants than commonly used SSM approaches, such as NNK codons, creating a more balanced library of mutations for screening [31]. We used a rational approach similar to that used to generate the superfolderGFP‐based Lime, targeting residues 145 and 146 based on the crystal structure of StayGold [17] (Figure S1). In Lime, the key residue S147D is characterized as adopting an inward‐facing side chain conformation that dramatically raises the p*K*_a_ of the chromophore (Figure 1a). We hypothesized that targeted mutations at the corresponding residues, which form hydrogen bonds with the chromophore in the structure of StayGold, would be most likely to lead to a similar inward‐facing conformation, thereby affecting the protein’s p*K*_a_ [17]. To mitigate potentially low signal‐to‐noise ratio in the initial prototype, we screened this library through standard brightness‐based colony selection, where fluorescing colonies were picked based on brightness from the agar plates and then evaluated based on their response to pH after growth in 96‐well plates (Figure 1b, left). We subsequently identified a variant with the mutations P145E and N146A with a Δ*F*/*F*_0_ of 2.03 from pH 5.5 to pH 7.4, and termed it serapH0.1.

Next, to expedite the evolution of serapH, we developed a novel method to overcome the limitations of traditional two‐step screening. In order to increase the screened library size in each round of evolution and consolidate the brightness and response‐based screening steps, we designed a system that incubates bacterial colonies directly on agar in a sealed chamber with CO_2_ (Figure 1d). We hypothesized that this would transiently acidify the cytosol in the bacterial colonies, highlighting the extent of pH‐dependent response in the proteins expressed within them. In addition to consolidating the brightness and response screenings into a single step (Figure 1b, right), this method substantially increases the number of colonies that can be feasibly screened in a single round.

**FIGURE 2:**
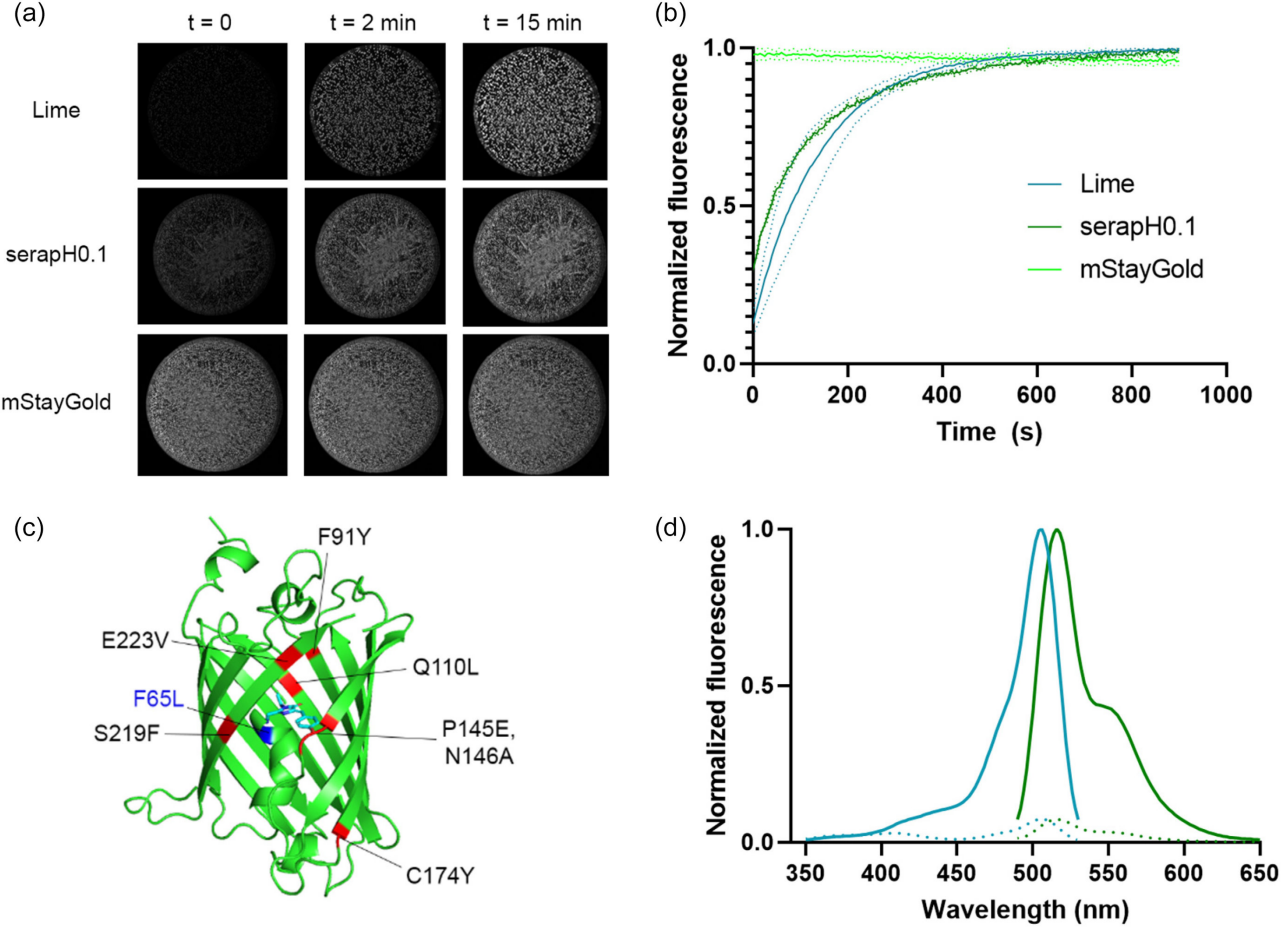
Gradual restoration of pH‐dependent fluorescence in agar colonies after CO_2_ incubation at 470 nm ex./510 nm em. Imaged data from agar plates (a) were captured every 5 s and processed in ImageJ to calculate normalized fluorescence over time (b) in *n* = 3 plates. Dotted lines represent error bars. (c) AlphaFold3 model [32] of serapH1.0 with mutations marked in red, and the chromophore of StayGold (PDB ID: 8BXT) in cyan, superimposed over 66Y. F65L, which was found during evolution but reverted to wild‐type in serapH1.0 and is therefore not present in the modeled protein, is marked in blue. (d) Excitation and emission spectra of the final evolved variant serapH1.0 (pH 7.4 in solid lines, pH 5.5 in dotted lines). The fluorescent response Δ*F/F*_0_ = 20.7 was calculated using the average emission from 500–540 nm at pH 5.5 and pH 7.4 using the formula Δ*F*/*F*_0_ = (*F*_pH7.4_ *–F*_pH5.5_)/*F*_pH5.5_.

To test our hypothesis, we placed a Petri dish containing a pH 7.1 10 mM Tris buffer inside the incubation chamber and incubated it with CO_2_. After 15 min of incubation, CO_2_ had reduced the buffer pH to 4.64 ± 0.13, a result we also confirmed visually with the indicator bromocresol purple. Next, we tested pH 7.4 agar plates with colonies expressing serapH0.1, mStayGold, and Lime. We found that serapH0.1 and Lime showed substantial and reversible fluorescent responses to CO_2_ incubation, whereas mStayGold showed little to no change (Figure 2a,b). This response reached a maximum after approximately 15 min of incubation, likely corresponding to the time required for CO_2_ to reach equilibrium within the cell, and returned to baseline 15 min after removal from the chamber. For ease of imaging, we opted to use the restoration of fluorescence to obtain data for screening pH‐dependent response. Notably, the fluorescent response of each protein on agar was substantially lower than previously reported in vitro, yet remained proportional to the responses of the other proteins. We also observed that, by imaging serapH with two filters at 436 nm ex./480 nm em. and 470 nm ex./510 nm em., we could quantify both fluorescence restoration in the 480 nm channel and fluorescence loss in the 510 nm channel, as a product of Tyr66 phenol‐phenolate equilibrium as pH returned to 7.4. After establishing the assay’s functionality and conditions, we used it as the basis for selection in several rounds of directed evolution on serapH.

After 10 rounds of random whole‐gene mutagenesis and CO_2_‐incubation‐based screening, serapH’s fluorescent response between pH 5.5 and pH 7.4 improved to 21‐fold. We initially generated libraries using mutagenic polymerase chain reaction (PCR) using a mix with a 0.1 mM concentration of manganese chloride, but found that among the best variants screened in the first three to four rounds of evolution (four top variants were selected each round), a majority were template. We addressed this by incrementally increasing the mutation rate until we observed fluorescence in only 50% of the total library per round. After this adjustment, we saw more substantial improvements in the pH‐dependent response with each round of mutation. Despite selecting for both brightness and response, we also observed a general decline in brightness from round to round. The resulting variant, serapH0.9, had eight mutations in total: two rational site‐directed mutations used to generate the initial prototype serapH0.1, and six random mutations discovered through evolution, one of which (F65L) was subsequently reverted, as described below (Figure 2c). In an effort to restore some of the original brightness, we finally performed a staggered extension process (StEP) to shuffle mutations between the evolved variant and the brighter serapH0.1 [33]. We reasoned that some of the random mutations identified during evolution may have had a minor effect on response but a significant effect on fluorescence, and aimed to determine whether reversing these mutations in any combination would restore brightness to the protein. Screening of the shuffled library identified a final variant in which F65L had reverted to the wild‐type phenylalanine (L65F), displaying 130% higher brightness and a response similar to that of the variant selected by evolution (Figure 2d). We denote F65L in Figure 2c because residue 65 lies immediately N‐terminal to the chromophore‐forming Tyr66, and the observation that a single substitution at this position accounts for a 130% difference in brightness at essentially unchanged pH response identifies it as a determinant of brightness in the mStayGold scaffold. We termed this variant serapH1.0 and characterized the protein in detail.

### 2.2 In Vitro Protein Characterization

**FIGURE 3:**
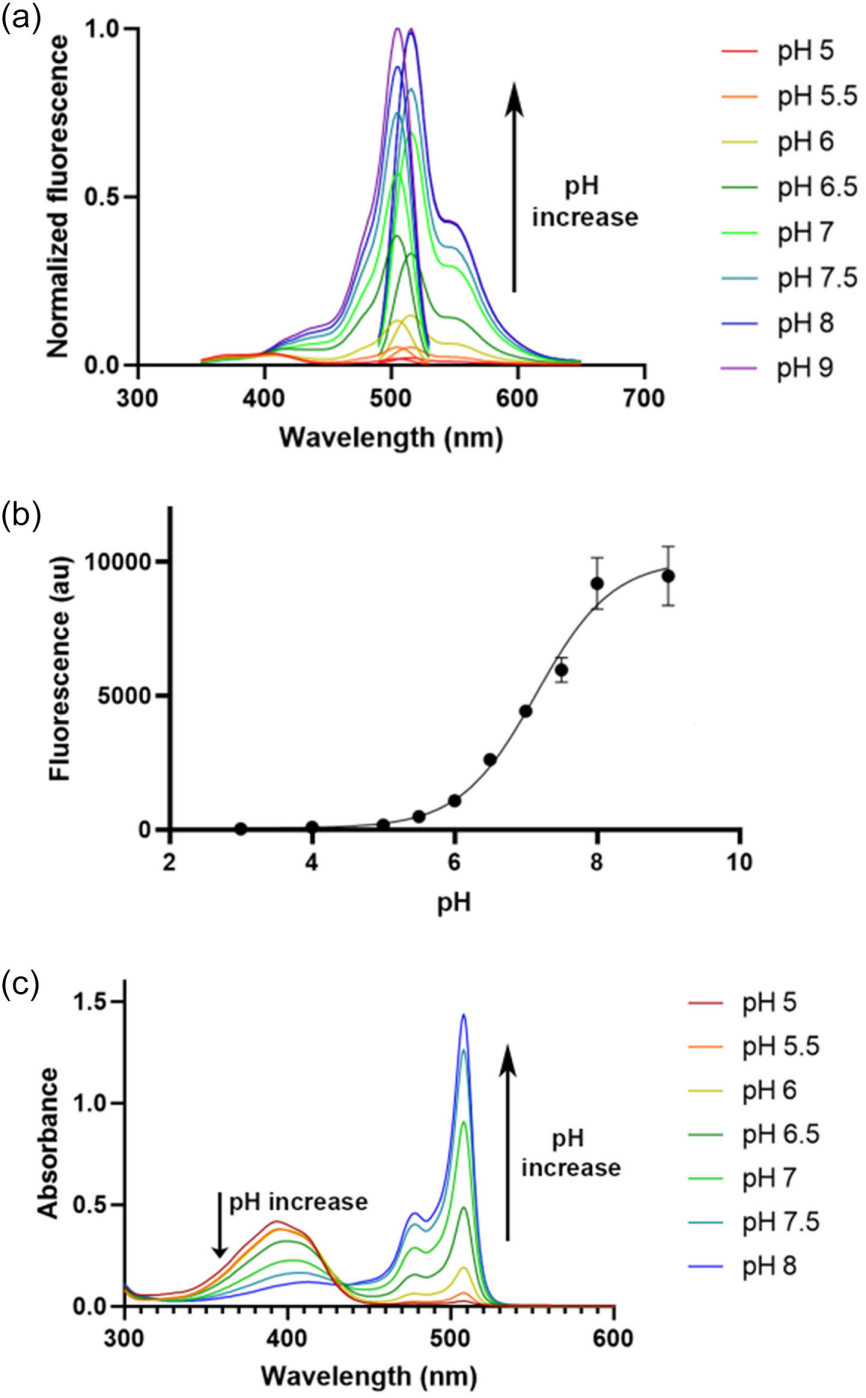
Spectral characterization of serapH1.0. (a) Emission and excitation spectra of serapH as a function of pH. The excitation spectra were taken by scanning from 350 to 530 nm with emission read at 575 nm, and the emission spectra were taken by scanning from 490 to 650 nm with excitation read at 455 nm. (b) pH titration curve of serapH1.0 using emission values averaged from 500 to 540 nm. p*K*_a_ = 7.1 and Hill coefficient n_H_ = 0.81 were calculated using a sigmoidal 4PL fit (GraphPad Prism). (c) Absorbance spectra taken by spectrometry at various pH values taken at 15 µM protein concentration.

We assessed the spectral characteristics of serapH to determine whether it would function adequately as a tool for imaging pH changes in living cells compared with existing sensors. To characterize the protein in vitro, we prepared large‐scale purification of serapH, Lime, SEP, and mStayGold. We selected Lime and SEP as the benchmarks for performance comparison due to their respective statuses as the brightest and most widely used GFP‐based pH sensors, and mStayGold for direct comparisons of photostability with the original scaffold of serapH. At pH 7.4, serapH1.0 exhibited an excitation maximum (*λ*_ex_) at approximately 506 nm and an emission maximum (*λ*_em_) of 516 nm (Figure 3a). This was slightly red‐shifted relative to the spectra of the original mSG protein, and its molecular brightness was also lower as a result of evolution. Between pH 5.5 and pH 7.4, we calculated the Δ*F*/*F*_0_ of serapH to be 20.7. Additionally, we performed a pH titration, which indicated a p*K*_a_ of 7.1 and a Hill coefficient n_H_ of 0.81 (Figure 3b). The quantum yield of serapH was determined to be unchanged between the high‐ ‍and low‐pH states, remaining at 0.94 (Table 1). We also determined the absolute molar extinction coefficient of serapH1.0 to be 110,000 M^−1^ cm^−1^. The molecular brightness of serapH, at 110 mM^−1^ cm^−1^ (EC * QY/1000), was slightly diminished from the original mStayGold (130 mM^−1^ cm^−1^) but remained higher than Lime (35 mM^−1^ cm^−1^). We also obtained absorbance spectra and observed two peaks at 398 and 508 nm, characteristic of the phenol and phenolate forms of Tyr66, respectively (Figure 3c). These characteristics showed suitability for assaying physiological pH, and to further distinguish serapH, we also addressed the key property of photostability that motivated our initial choice of mStayGold.

**TABLE 1:** Spectral characteristics of serapH and controls. Characterized data for Lime and mStayGold were obtained from their respective publications, except for *t*_1/2_, which was measured experimentally.

	Excitation maximum, nm	Emission maximum, nm	**Extinction coefficient,** **EC, M** ^**−1**^ **cm** ^**−1**^	**Quantum yield (QY), λ**_**ex**_ ** = 499 nm**	**Mol. brightness,** EC*QY/1000, mM ^**−1**^ **cm** ^**−1**^	**p*K***_***a***_	***t***_**1/2**_ **in widefield oil suspension, s**
Lime [17]	488	510	45,000	0.77	35	7.1	52
SEP	488	511	37,000	0.80	30	7.0	174
mStayGold [29]	499	510	160,000	0.83	130	4.8	259
serapH1.0	506	516	110,000	0.94	100	7.1	242

### 2.3 Characterization of Photostability

**FIGURE 4:**
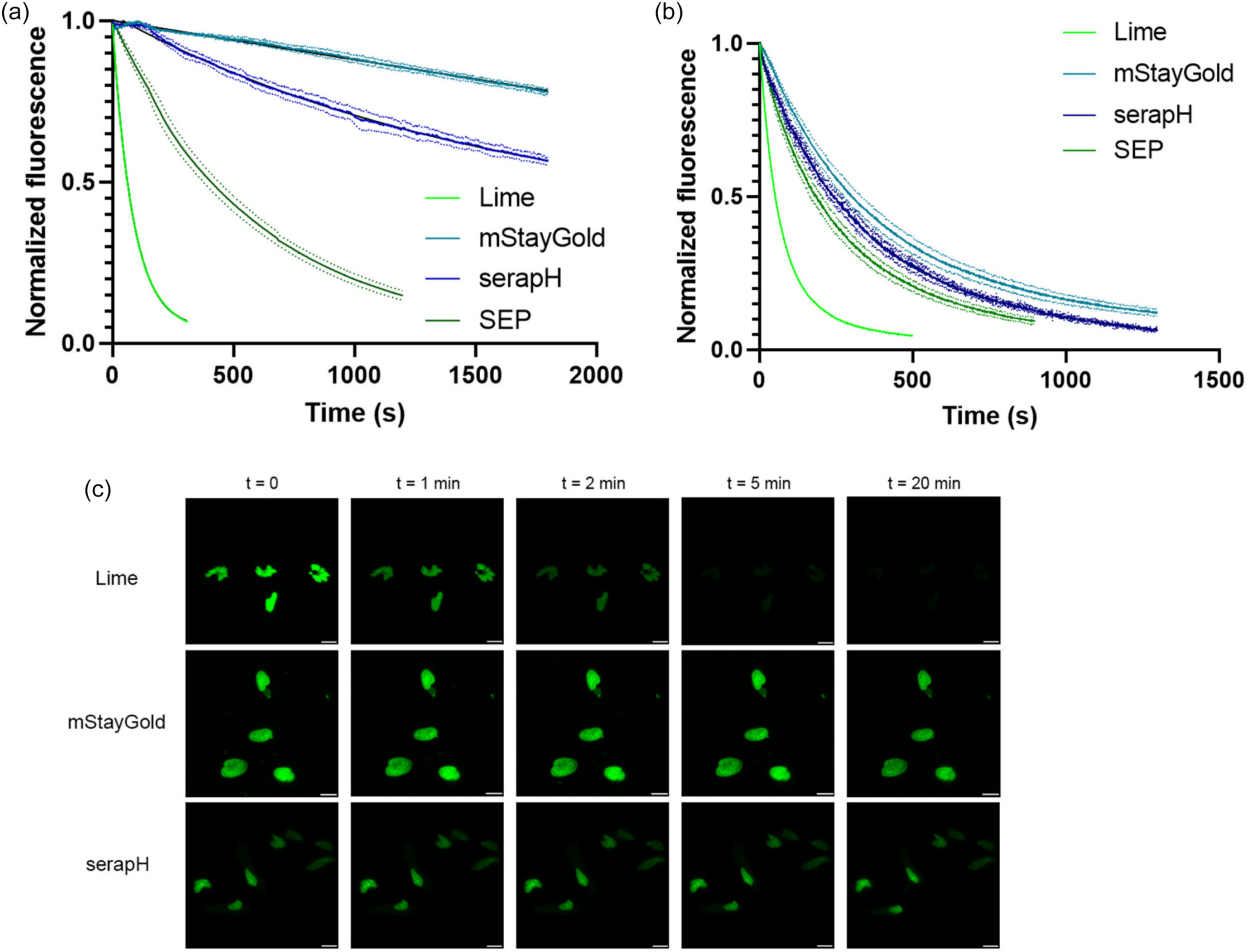
Photostability measurements of serapH and controls. Photostability was assayed through continuous illumination of the protein in a histone 2B fusion in HeLa cells (a) or in purified protein suspended in mineral oil (b) at 488 nm excitation and 518 nm emission. Curves shown represent three biological replicates (*n* = 3 independent experiments). Both assays were performed using widefield microscopy with 488 nm excitation light at an irradiance of approximately 4.8 mW/cm^2^. (c) Representative time‐lapse of photobleaching in H2B fusions. Scale bars represent 20 µm.

Since we selected mStayGold as a scaffold for its high photostability relative to existing GFP‐based sensors, we directly compared the photostability of evolved serapH with existing benchmarks to see if photostability had been retained during directed evolution. We evaluated photostability in two ways: by illumination of purified protein solutions suspended as droplets in mineral oil, and by illumination of HeLa cells expressing the protein as a fusion to histone 2B. Using measurements from imaging protein droplets in mineral oil, we observed that the photobleaching half‐life (*t*_1/2_) of Lime was 52 s, while serapH and mStayGold were similar at 242 and 259 s, respectively (Table 1). SEP also displayed surprisingly high photostability, with a *t*_1/2_ value of 174 s, but still remained less photostable than serapH. We observed even greater differences in photostability in the H2B fusions in mammalian cells, where serapH and mStayGold exhibited only minor photobleaching over 25 min under our excitation conditions (Figure 4a,b). In both cases, serapH displayed photostability that was slightly lower than that of mStayGold, but still much higher than that of Lime or SEP.

### 2.4 Imaging of WPB Exocytosis

**FIGURE 5:**
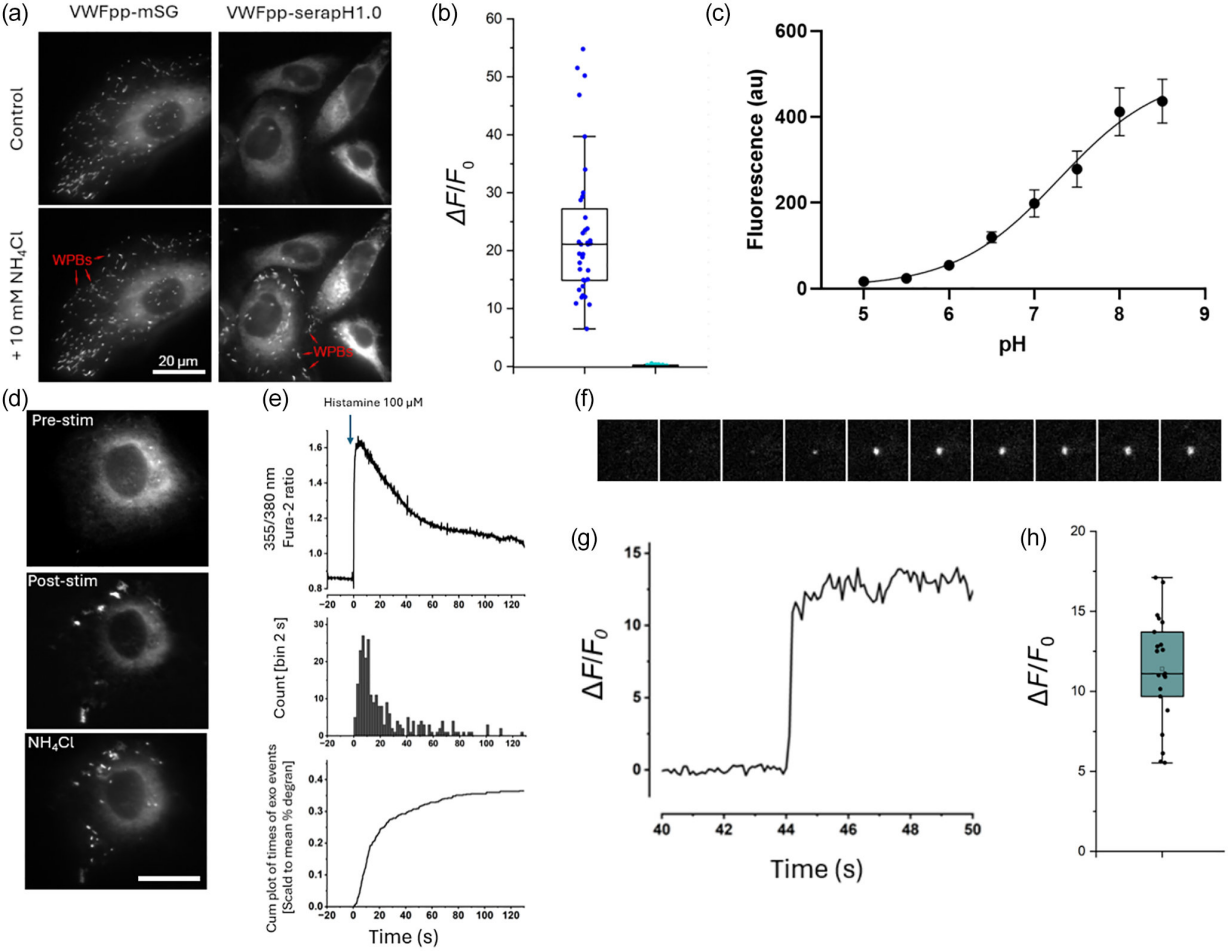
Imaging WPBs in HUVEC using a serapH‐VWFpp construct. (a) HUVEC expressing VWFpp‐serapH and mSG before and after alkalinization with NH_4_Cl. Examples of VWF‐containing WPBs are labeled with red arrows. (b) Fluorescent response (Δ*F*/*F*_0_, or (*F*‐*F*_0_)/F_0_) of serapH1.0 (blue, left) and mSG (cyan, right) in WPBs before and after alkalinization (36 WPBs, four cells, mean 23.1 ± 2.0 fold for serapH1.0; 23 WPBs, three cells, mean 0.193 ± 0.11 fold for mSG). (c) pH titration of serapH1.0 in HUVEC. p*K*_a_ = 7.3 and n_H_ = 0.72 were calculated as described in Figure 3. (d) Representative images of HUVEC expressing VWFpp‐serapH1.0 before and after histamine‐induced stimulation. Alkalinization reveals the remaining population of WPBs that have not undergone exocytosis. (e) Kinetics of histamine‐induced WPB exocytosis events (244 WPBs, 11 cells). (f) Representative time course of an individual exocytosis event at 10 frames per second, with an observed Δ*F*/*F*_0_ of ~13‐fold (shown in (g)). (h) Averaged data (21 WPBs, five cells) showing a mean Δ*F*/*F*_0_ of 11.4 ± 0.73 fold.

We next tested the performance of serapH1.0 in a cell‐based application by using it to monitor the exocytosis of WPBs in human umbilical vein endothelial cells (HUVEC). WPBs store the hemostatic protein VWF, which is released into the extracellular environment in response to stimulation [3, 4]. WPBs initially form at the trans Golgi network with an intraluminal pH of 6‐6.5, but rapidly acidify the lumen to between pH 5−6 on maturation. Following WPB‐plasma membrane fusions, there is a rapid shift in WPB luminal pH to that of the extracellular environment [34]. To monitor this shift, we first generated a construct of serapH1.0 fused to the C‐terminus of the VWF propeptide (VWFpp) [15]. Immunofluorescence staining of HUVEC 24 h post‐transfection with VWFpp‐serapH1.0 showed robust expression and colocalization with mature VWF in WPBs (Figure S4). To test the pH sensitivity of WPB‐targeted VWFpp‐serapH1.0, we compared the effect of acute WPB alkalinization with the weak base NH_4_Cl on WPB fluorescence intensity (FI) in cells expressing VWFpp‐serapH1.0 and the pH‐insensitive mSG variant, VWFpp‐mSG [3]. Expression of VWFpp‐mSG in living HUVEC showed a diffuse perinuclear fluorescence, corresponding to the endoplasmic reticulum [34] (ER; which in these cells has a luminal pH of ~7.2), and abundant rod‐shaped WPBs distributed through the cytosol (Figure 5a). VWFpp‐serapH1.0‐expressing HUVEC also showed ER labeling with occasional rod‐shaped structures that most likely correspond to immature WPBs. As expected, addition of NH_4_Cl to VWFpp‐mSG‐expressing HUVEC produced little or no increase in WPB FI. In contrast, treatment of VWFpp‐serapH1.0‐expressing HUVEC with NH_4_Cl produced the dramatic appearance of mature WPBs that had previously been undetectable. The dynamic range of serapH was approximately 23‐fold, consistent with in vitro characterization of the fluorescent response (Figure 5b). To quantify the pH dependence of serapH1.0 fused to VWFpp in living HUVEC, we carried out an in‐cell pH titration that yielded an apparent p*K*_a_ of 7.3 and an n_H_ of 0.72. The results show that the in vitro properties of serapH1.0 are largely retained in cells after fusion to VWFpp (Figure 5c).

We next tested the performance of serapH1.0 during WPB exocytosis. To trigger WPB exocytosis, VWFpp‐serapH‐expressing HUVEC were stimulated with 100 µM histamine. Histamine‐induced WPB exocytosis was revealed as discrete, abrupt increases in fluorescence within the cell cytosol (Figure 5f‐h, Supporting Videos). Addition of NH_4_CL at the end of the period of simulation (~2 min) revealed the non‐exocytosed population of fluorescent WPBs. Quantification of the responses showed a long delay, approximately 3 s, between the histamine‐evoked rise in intracellular free calcium ion concentration in the cell and the first exocytosis event, and that on average, 30%–40% of fluorescent WPBs underwent exocytosis within 2 min of stimulation, similar to previous data obtained using VWFpp‐EGFP as the reporter [34] (Figure 5d, e). The mean Δ*F*/*F*_0_ value observed from *n* = 21 individual WPB exocytosis events was 11.4 ± 0.73 (Figure 5h).

## 3 Discussion

Here, we have reported the development of serapH1.0, a highly photostable pH sensor based on mStayGold [29], and demonstrated a new methodology for selecting and evolving FP‐based pH sensors. SerapH1.0 has high molecular brightness, with 100 to 35 mM^−1^ cm^−1^ of Lime. With a p*K*_a_ of 7.1, it is closely aligned with existing pH sensors, such as SEP at 7.2 and Lime at 7.1. Most importantly, serapH overcomes the fundamental limitation of low photostability inherent in GFP‐based sensors. Our demonstration of serapH’s photostability showed *t*_1/2_ = 242 s to bleach to half the original fluorescence in our protein droplet assay, versus 52 s for Lime. We observed even higher photostability in HeLa cells with H2B fusions, which may be attributed to differences in the redox state of the two environments, or additional photon absorbance from cell membranes in the H2B fusions. We also demonstrated the effectiveness of serapH for visualizing exocytosis using HUVEC and WPB exocytosis as a model system. SerapH fused to the VWFpp was correctly targeted to WPBs, where it retained its pH sensitivity, allowing clear detection of hormone‐triggered exocytosis events. With a mean Δ*F*/*F*_0_ of 11.4 for exocytosis events, and a mean Δ*F*/*F*_0_ of 23.1 after alkalinization, serapH is well‐suited for imaging exocytosis in physiological or neuronal applications. With its resistance to photobleaching, serapH should be able to withstand longer time‐lapse image acquisition while supporting higher excitation intensities and lower gain. These qualities make it particularly well‐suited for applications that demand continuous imaging at relatively low expression levels [35]. They also expand the possibilities for visualizing intracellular protein trafficking, as they enable the use of super‐high‐resolution microscopy techniques [28, 29] that were previously not feasible due to the high risk of photobleaching [19].

Although we lack a crystal structure to explain the p*K*_a_‐altering mechanism behind the mutations we discovered over the course of developing serapH1.0, there is a plausible explanation based on the structural characterization of Lime. In Lime, the mutation S147D was found to have inverted orientation, with the polar side chain facing into the β‐barrel rather than outwards [17]. It is hypothesized that direct hydrogen bonding interactions between the chromophore and this aspartic acid residue are the primary cause for the raised p*K*_a_ of Lime relative to its scaffold (Figure S1). We used this as the basis for our own SSM of residues 145 and 146, which led to the discovery of the P145E mutation in serapH0.1. A similar interaction between this glutamic acid residue and the mStayGold chromophore is likely present in serapH and has also been further tuned by the additional mutations to the β‐barrel found through evolution. Likewise, N146A, which was discovered as a paired mutation with P145E, may enhance the effect of the glutamic acid residue by increasing the accessible space in the chromophore region and enabling a stronger interaction between the chromophore and E145.

The CO_2_‐based selection assay we have demonstrated is also an improvement to the directed evolution workflow of pH‐sensitive FPs. The benefit of this system is twofold: we can directly screen proteins for both pH response and brightness in agar colonies, thereby scaling the size of the screened library while efficiently evaluating protein functionality in a single step. Simultaneously evaluating brightness and response also allowed us to easily optimize the mutation rate during library generation. Compared to other high‐throughput screening methods such as FACS, our CO_2_ incubation method is relatively low‐tech and low‐cost, requiring only an airtight chamber to house agar plates and a light source and camera to capture fluorescence emission from the colonies. Using this method, the time and labor required per round for a pH‐sensitive FP could be reduced while increasing the likelihood of identifying positive mutations within each library.

Like other screening methods using crude lysates, the CO_2_ assay often conflates expression level with brightness when assessing colony fluorescence on agar. This is evidenced by the loss of molecular brightness in serapH (100 mM^−1^ cm^−1^) relative to mSG (130 mM^−1^ cm^−1^), despite continuous selection for both brightness and response across evolutionary rounds. We observed reduced fluorescent response for proteins in colonies compared to in vitro, which was consistent and proportional in both the controls and mutagenic libraries. One potential cause may have been the CO_2_’s inability to penetrate the full depth of the colony, leading to some cells being inadequately acidified. Visual artifacts in edge and low‐brightness colonies also tended to overrepresent them as more responsive, which we mitigated by applying a brightness threshold in subsequent rounds. Though we were able to address these issues, mainly by employing a confirmation assay in which selected colonies are expressed at a larger scale, they limit the CO_2_ assay from fully reflecting a library’s pH sensitivity and brightness.

While its current performance is already adequate for cellular imaging, serapH still shows room for improvement in both brightness and response compared with other green‐fluorescent pH biosensors. In addition to its diminished molecular brightness, serapH’s 21‐fold fluorescent response remains modest in comparison to its GFP‐based counterparts, which have demonstrated response levels upwards of 60‐fold. The mechanistic reason for this may be reflected in the difference in the apparent Hill coefficients between these sensors, which describes the rate of fluorescence change as pH shifts near the p*K*_a_ threshold. The Hill coefficient of serapH is *n*_H_ = 0.81, which is substantially lower than the 1.9 of SEP and 1.6 of Lime. The abnormally high *n*_H_ values of SEP and Lime suggest that, in going from low to high pH, there may be a secondary deprotonation event that acts cooperatively with the deprotonation of the chromophore [19]. Since our prototype‐generation strategy followed Lime’s rationale, which in turn used mutations from SEP, it stands to reason that serapH could also gain this cooperative interaction through further evolution and optimization. The development of other FP‐based biosensors suggests that significant increases in molecular brightness without sacrificing response can be achieved through further evolution [36, 37], which would strengthen serapH in applications requiring minimally disruptive expression, such as protein tagging [11, 35]. It is also important to note that while the molecular brightness of serapH is diminished relative to the original mStayGold, it is still substantially higher than Lime, meaning its brightness is not practically limiting for any existing application of Lime/SEP. To further improve the brightness and response of serapH, we plan to explore adjustments to the screening process, such as expansions to the incubation setup to incubate and image several plates in parallel. We also aim to perform more extensive characterization of serapH by evaluating its comparative performance in cells with SEP and Lime. Particularly, in‐cell pH titration and exocytosis assays under the same settings should provide a clear reference for the baseline pH of the cells, as well as a benchmark for serapH’s dynamic response in comparison to best‐in‐class GFP sensors.

With the performance we observed in cellular assays of exocytosis and photobleaching, we have demonstrated the functionality of serapH as a highly photostable pH biosensor. Likewise, the pH‐sensitive FP screening system we have demonstrated addresses some of the limitations of standard screening methods. We aim to apply the methodology developed here to further improve serapH and other relevant pH‐sensitive proteins. Ultimately, we believe serapH will offer new insights into the dynamics of membrane protein trafficking to intracellular compartments, particularly through the enabling of super‐resolution imaging modalities previously inaccessible due to the photostability limitations of GFP‐based sensors.

## 4 Methods

### 4.1 General Materials

Synthetic oligonucleotides were purchased from Thermo Fisher Scientific. The pBAD base plasmid encoding mStayGold was obtained as a gift from the Miyawaki research group. Phusion High‐Fidelity DNA Polymerase (Thermo Fisher Scientific) was used for routine PCR amplification and single‐site‐directed mutagenesis, while Taq DNA Polymerase (New England Biolabs) was used for error‐prone whole gene mutagenesis. GeneJET Miniprep and Midiprep Kits were purchased from Thermo Fisher Scientific. Bacteria were transformed by electroporation (Bio‐Rad, 1652100). Proteins were expressed in *E. coli* DH10B cells (Thermo Fisher Scientific) in LB media supplemented with 100 μg/mL ampicillin and 0.02% L‐arabinose, and extracted using bacterial protein extraction reagent (B‐PER) (Thermo Fisher Scientific) unless otherwise noted. Fluorescence excitation and emission spectra were recorded on a Spark plate reader (Tecan). Data were represented and analyzed using GraphPad Prism. Error for measurements is provided as mean ± standard deviation (SD) unless otherwise stated.

### 4.2 Rational Engineering and Directed Evolution of serapH from mStayGold

To generate the initial serapH prototype, we applied 22C mutagenesis to two residues near the chromophore. This library was screened by fluorescence on agar plates (50 µg/mL ampicillin, 0.02% w/v L‐arabinose), and 192 fluorescent colonies were manually selected and grown in 1 mL of Terrific Broth (TB, 50 µg/mL ampicillin, 0.02% w/v L‐arabinose) in 96‐deep‐well dishes overnight. Cultures were centrifuged at 4,000 × g at room temperature, then extracted with 50 μL of B‐PER (Thermo Fisher Scientific). A 10 µL of the B‐PER extract was added to a 96‐well plate and diluted with 90 µL of pH 7.4 buffer (50 mM HEPES, 50 mM MES, 200 mM NaCl) or pH 5.5 buffer (50 mM HEPES, 50 mM MES, 200 mM NaCl), after which the emission was measured using the plate reader. Excitation was measured from 350 to 530 nm with emission read at 575 nm, while emission was measured from 490 to 650 nm with excitation read at 455. A variant was selected from this screening based on fluorescent response from pH 5.5 to pH 7.4 for use as serapH0.1.

For directed evolution, the prototype was subjected to whole‐gene random mutagenesis, using error‐prone PCR with Taq polymerase and MnCl_2_ at a concentration tuned to generate approximately one to two mutations in the 800 bp gene. Blunt‐end DNA fragments containing the mutant serapH gene were generated using this method. The PCR products and pBAD vector were then digested with restriction enzymes (Thermo Fisher Scientific) to yield gene fragments with sticky ends, which were subsequently ligated, cleaned, and concentrated (Zymo Research) to create a serapH library with random mutations. The libraries were transformed into DH10B *E. coli* and plated on agar plates (100 µg/mL ampicillin, 0.02% w/v L‐arabinose), which were then incubated at 37 °C for 12–16 h to allow adequate colony growth and expression.

Each plate was placed in a box and incubated with CO_2_ by feeding gaseous CO_2_ from a jar containing dry ice (Figure 1) for 15 min. After lowering the pH of the cellular environment, the plates were removed and imaged at 1 frame per 30 s for 15 min using Micro‐Manager and an arc lamp (Sutter Instruments) with a 470 nm ex./510 nm em. filter to monitor the restoration of fluorescence as CO_2_ equilibrated and the pH returned to baseline. The captured images were analyzed using ImageJ’s threshold function to create a mask highlighting each colony, and the region of interest (ROI) function was used to calculate their fluorescent response based on brightness values at the start and end of the elapsed time. Using these images, four to six colonies in each round with the highest brightness and response were selected. Selected colonies were picked and cultured in 4 mL TB (100 µg/mL ampicillin, 0.02% ‍w/v L‐arabinose) and then extracted with 100 µL B‐PER. 10 µL of the B‐PER extract was assayed with 90 µL of each of the previous pH 5.5 and pH 7.4 buffers in 96‐well optical plates (Thermo Fisher Scientific). The best‐performing variant based on both brightness and response was selected and used as the template for mutagenesis in the next round.

After 10 rounds of evolution, the initial and final variants of serapH were combined to generate a library using the staggered extension process (StEP). The initial serapH0.1 prototype with two mutations and the final serapH variant evolved from it were combined into a PCR mix at 20 ng each, and a modified PCR protocol was performed with no extension step for 100 cycles [33]. PCR products were separated by agarose gel electrophoresis, and the band corresponding to the expected gene size was excised, extracted, digested, and ligated into a pBAD expression vector. This library was screened using the CO_2_ box described above to see if any combination of reverted mutations could yield a better variant. A variant was identified with a single reverse mutation that maintained a high response while restoring significant brightness, and we selected it for use as the finished sensor serapH1.0.

### 4.3 Protein Purification by Nickel‐NTA

After transforming *E. coli* DH10B with pBAD plasmids encoding the protein of interest carrying an N‐terminal 6×His‐tag, colonies were picked and used to inoculate a 5 mL LB (50 µg/mL ampicillin) seed culture at 37 °C and 200 rpm overnight. The starter cultures were added to 250 mL of TB (100 µg/mL ampicillin). TB cultures were shaken at 250 rpm and 37 °C until OD600 was approximately 0.6, and then induced with a final concentration of 0.02% w/v L‐arabinose at room temperature and 200 rpm overnight. Cultures were pelleted at 4,000 × g, resuspended in lysis buffer (50 mM phosphate buffer, 300 mM NaCl, 1% B‐PER, pH 8), and lysed by sonication with a Branson sonicator. The whole cell lysate was centrifuged at 12,000 × g and 4 °C for 30 min to pellet cell waste, and the supernatant was loaded onto 1 mL of nickel‐NTA resin (Wako). The cell lysate was then incubated with the resin at 4 °C with rotation for 2 h. The resin was washed with wash buffer (50 mM phosphate buffer, 300 mM NaCl, 20 mM imidazole, pH 8) for 3 × 5 resin volumes (RVs). Following this, the protein was eluted from the beads with elution buffer (50 mM phosphate buffer, 300 mM NaCl, 250 mM imidazole, pH 8), and the eluate was collected in a Falcon tube. After purification, the imidazole was removed by exchanging into storage buffer (10 mM Tris, 150 mM NaCl, pH 7.3) using a 10 kDa MWCO Amicon centrifugation filter.

### 4.4 In Vitro Characterization of serapH

For characterization of excitation and emission spectra, proteins were assayed at 1 µM in pH 5.5 and pH 7.4 buffers in a 384‐well optical plate (Thermo Fisher Scientific). Excitation was measured from 350 to 530 nm with emission read at 575 nm. Emission was measured from 490 to 650 nm, with excitation at 455 nm. To calculate the Δ*F*/*F*_0_, emission values between 500 and 540 nm (step size = 2, 20 values) were averaged from *n* = 3 wells for samples at pH 5.5 and pH 7.4, and calculated from the formula Δ*F*/*F*_0_ = (*F*_pH7.4_‒*F*_pH5.5_)/*F*_pH5.5_. Spectra were obtained from a single protein preparation with three technical replicates.

For the pH titration, a series of buffers was prepared using 30 mM trisodium citrate, 30 mM sodium borate, 30 mM MOPS, and 100 mM KCl and adjusted to pH values ranging from 3 to 11. The pH titration was performed with a single protein preparation and three technical replicates. Emission spectra at each pH value were obtained according to the characterization protocol described above and averaged into a single value. The apparent p*K*_a_ of serapH1.0 was found by using a sigmoidal 4PL fit curve (GraphPad Prism) applied to the plotted values of fluorescence and pH and calculating the pH value found at the inflection point of the fitted curve. The Hill coefficient n_H_ was calculated using the same fitted curve by taking the linear slope found at the inflection point. Absorbance spectra were measured using the Shimadzu UV1800 spectrometer from 300 to 650 nm for proteins in the above buffer series at a protein concentration of 15 µM. Absorbance spectra were taken using a single protein preparation with three technical replicates for each pH value.

Absolute molar extinction coefficients were calculated using previously reported methods to maintain parity with the original characterization of mStayGold [29]. Protein was prepared at 10 µM concentration in pH 7.4 buffer, and absorbance spectra were taken from 300 to 650 nm using a Shimadzu UV1800 spectrometer. The same samples were then recovered from the cuvette and denatured using NaOH at a final concentration of 0.1 M. The denatured protein solution was scanned again using the same conditions, and the absorbance ratio of the largest peak of each spectrum was used with the molar extinction coefficient of the denatured chromophore structure, 44,000 M^−1^ cm^−1^, to calculate the absolute molar extinction coefficient of the protein. Fluorescence quantum yield was measured with 10 µM of protein in a pH 5.5 or pH 7.4 buffer using a Hamamatsu Photonics absolute quantum yield spectrometer (C9920‐02G), at an excitation wavelength of 499 nm. Quantum yield and extinction coefficient measurements were both carried out in biological triplicate (three separate protein preparations) with three technical replicates per condition.

### 4.5 Photostability Characterization of serapH

Photostability of serapH was evaluated both in oil droplets containing purified protein and in HeLa cell nuclei expressing the protein. An IX83 wide‐field fluorescence microscope (Olympus), using a pE‐300 LED light source (CoolLED) and a 40× objective lens (numerical aperture (NA) = 1.3; oil), an ImagEM X2 EMCCD camera (Hamamatsu), and Cellsens software (Olympus) were used for all microscopy experiments. Imaging was conducted using 488 nm ex./518 nm em. filters. The LED laser was measured using a Hioki 3664 optical power meter to confirm an intensity of 4.8 mW at the staging area. All photostability measurements were performed in biological triplicate with the average of *n* = 3 cells per experiment for three technical replicates. The mean fluorescence value of each biological replicate, and the SD of the three replicates, was plotted in GraphPad Prism.

### 4.6 Purified Protein Droplets

Assaying of purified protein in mineral oil was done according to previously reported methods [38] in order to compare photobleaching between benchmark FPs and serapH1.0. To neutralize the pH of the mineral oil for the protein suspension, a simple extraction was performed using a pH 7.3 Tris buffer. Briefly, 1 mL of mineral oil was vortexed with an equal volume of buffer, allowed to settle, and the buffer was replaced manually by pipette. This process was repeated three to four times until the buffer was no longer cloudy after vortexing. Purified protein was diluted in pH 7.4 or pH 5.5 buffer (5 µM for pH 7.4, and 50 µM for pH 5.5 to compensate for low fluorescence at low pH; except mSG, which was assayed at 10 µM at pH 5.5 due to its relative stability at low pH), and then suspended in a 1:100 ratio in neutralized mineral oil to a total volume of 100 µL. This mixture was vortexed at high speed for 30 s to disperse the aqueous protein droplets, and then 10 µL was applied to a coverslip and imaged continuously at 1 frame per second for 30 min. Image stacks were analyzed using ImageJ to highlight individual protein droplets. Fluorescence of *n* = 3 individual droplets was averaged in each frame and plotted over time, and a single‐decay exponential function was applied to calculate the half‐life *t*_1/2_ for each protein. This process was repeated for three separate protein preparations, for *n* = 3 biological replicates.

### 4.7 Cell Imaging with Histone 2B Fusions

pcDNA plasmids containing histone H2B‐serapH, Lime, and mSG were created by Gibson assembly (New England Biolabs) using the mPapaya1‐H2B‐6 plasmid as a backbone (Addgene #56651). HeLa (American Type Culture Collection; ATCC #CCL‐2) cells were cultured in Dulbecco’s modified Eagle medium (DMEM high glucose; Nacalai Tesque) supplemented with 10% fetal bovine serum (FBS; Sigma–Aldrich) and 100 μg/mL penicillin and streptomycin (Nacalai Tesque). Cells were seeded in 35 mm glass‐bottom dishes (IWAKI). The next day, cells were transfected with pcDNA‐H2B‐serapH, Lime, or mSG using polyethyleneimine (PEI, Polysciences) in Opti‐MEM I medium. 4 h after transfection, the Opti‐MEM I solution was aspirated and replaced with 2 mL of warmed culture medium. The cells were then imaged 48–72 h after transfection. Before imaging, the culture medium was aspirated, washed with Hank’s balanced salt solution (HBSS, Nacalai Tesque), and exchanged into HBSS. Cells expressing H2B‐serapH and H2B controls were imaged continuously for 30 min and analyzed using ImageJ. Fluorescence of *n* = 3 individual nuclei was averaged in each frame and plotted over time. This process was repeated with cells prepared from three separate passages, for *n* = 3 biological replicates.

### 4.8 Imaging in HUVEC Using serapH

#### 4.8.1 Tissue Culture, Transfections, and Immunocytochemistry

HUVEC were purchased from PromoCell (#C12203, GmbH, Heidelberg, Germany) and cultured (maximum passage, P4) in human growth media (HGM) comprising Medium 199 Earle’s salts + L‐Glutamine (Cat. No. 11150059, Thermo Fisher), supplemented with 20% fetal calf serum, 30 µg/mL Endothelial Cell Growth Supplement (Upstate: Cat. No.: 02‐102), 10 U/mL heparin (Sigma–Aldrich: Cat. No.: H‐3149) and 50 µg/mL gentamicin (Invitrogen: Cat. No. 15750‐037) at 37 °C in a 5% CO_2_ atmosphere. HUVEC were nucleofected with 2–4 μg of expression vector DNA; VWF‐propeptide‐EGFP [14] (VWFpp‐EGFP), VWFpp‐mSG [15], or VWFpp‐serapH1.0 using the Amaxa Nucleofection device (Lonza Cologne AG) according to the manufacturer’s instructions. Transfected cells were plated at confluent density in complete HGM onto 35 mm diameter poly‐lysine‐coated glass bottomed culture dishes (MatTeK Corp., Ashland, USA) for live cell imaging of WPB exocytosis and in‐cell pH calibrations, or onto 1% porcine gelatin (Sigma‐Aldrich; G1890) coated 9 mm diameter glass coverslips for immunocytochemistry. Cells were used 24–48 h post‐transfection.

Immunocytochemistry was performed on 3% paraformaldehyde‐fixed and 0.2% saponin (Sigma–Aldrich, S7900) permeabilized cells as previously described [16]. Antibodies used were a sheep anti‐VWF antibody (clone AHP062 at 1:8000, Serotec Raleigh, NC, USA) and a rabbit monoclonal anti‐VE‐Cadherin (#2500C, 1:250, Cell Signalling Technology, 2316 WZ Leiden, NE). Coverslips were viewed using a 60 × 1.4 NA oil objective on a Bio‐Rad Radiance 2100 confocal microscope. Images were acquired sequentially (488 nm, 542 nm) at 1024 × 1024 pixels with a 6‐frame Kalman average using Bio‐Rad Radiance software. Files were exported to ImageJ/Fiji (v1.54p) or Adobe Photoshop CC release 23.2.2. Image analysis was carried out in Winfluor (http://spider.science.strath.ac.uk) or ImageJ/Fiji as previously described (Bierings et al., 2012). Dataset plotting, fitting, and analysis were performed in Origin 2025 (64‐bit) SR1 10.2.0.196 (Academic) (OriginLab Corporation). Results were expressed as mean ± standard error of the mean (SEM).

### 4.9 Cellular pH Titration

In‐cell pH calibrations of VWFpp‐serapH1.0 were carried out using previously described methods [21]. HUVEC transfected with VWFpp‐serapH1.0 were seeded onto 35 mm glass‐bottomed dishes and cultured for 24 h. For live cell experimentation, cells were transferred from HGM to a physiological saline solution (140 mM NaCl, 5.4 mM KCl, 2 mM MgSO_4_, 1.8 mM CaCl_2_, 10 mM HEPES, 8 mM glucose, pH 7.3). Immediately before experimentation, the pH calibration solutions (120 mM KCl, 20 mM NaCl, 0.5 mM CaCl_2_, 0.5 mM MgSO_4_, and 20 mM HEPES, pH adjusted between pH 8.5–5.0 in 0.5 pH steps using KOH or HCl) were supplemented with 10 µM carbonyl cyanide m‐chlorophenylhydrazone (CCCP; Sigma–Aldrich, C2759‐250 MG) and 7.5 µM nigericin [21, 34] (Sigma–Aldrich, N7143‐5 MG) and the cells washed once in the calibration solution before being incubated in fresh calibration solution in the dark for 5–7 min at room temperature, before imaging. Cells were imaged on an Olympus IX71 inverted fluorescence microscope equipped with an Olympus UAPO N 340 40 × 1.35 NA oil immersion objective. Excitation light (488 ± 5 nm) was from an Optoscan monochromator (Cairn Research, Faversham, UK), and emission light (525 ± 20 nm) was captured using a back‐thinned Andor IXon3 EMCCD camera (Oxford Instruments, UK) operated in frame transfer mode at full gain (exposure time 200 ms) using Winfluor software (http://spider.science.strath.ac.uk). Multiple fields of view were recorded for each pH experiment. Experiments were repeated with *n* = 3 biological replicates using the data of all VWFpp‐serapH ROIs in *n* = 3 cells per replicate. Data across the three replicates were averaged and plotted with SD in GraphPad Prism, and fitted with the same sigmoidal 4PL model as the in vitro titration.

### 4.10 Live Cell Imaging of Fura‐2 Fluorescence and Fluorescent WPB Exocytosis

Epifluorescence imaging of changes in intracellular free calcium ion concentration ([Ca^2+]^ *_i_*) and fluorescent WPB exocytosis in Fura‐2‐loaded (1 µM Fura‐2/AM, Thermo Fisher #F1225, for 20 min in the dark at room temperature) HUVEC was carried out as previously described [34]. Cells were stimulated to undergo WPB exocytosis using histamine (100 µM), and to quantify the fraction of fluorescent WPBs that had undergone exocytosis, non‐exocytosed WPBs were revealed by acute application of the weak base ammonium chloride (NH_4_Cl; 10 mM) at the end of the period of stimulation. Data for kinetics and fluorescence change were all obtained in single independent experiments, with *n* = 4 cells for the alkalinization experiment, *n* = 11 cells for the kinetics experiment, and *n* = 5 cells for the exocytosis fluorescence change experiment. Results were expressed as mean ± standard error of the mean (SEM).

## Author Contributions

M.C. performed directed evolution and in vitro characterization of serapH. M.C. and K.K.T. designed and constructed the CO_2_‐based screening system. M.C., K.T‐Y., K.K.T., and R.E.C. designed and performed imaging‐based photostability experiments. T.C., R.B., and L.N. designed and conducted pH titration and exocytosis experiments in cells. R.E.C., K.K.T., and M.C. conceived the project premise and rationale for creating an initial serapH prototype. M.C., K.K.T., T.T., R.B., T.C., and R.E.C. wrote the manuscript. K.K.T., T.T., and R.E.C. supervised research.

## Funding

This study was supported by the Japan Society for the Promotion of Science (19H05633, 22H04743, 24H02267, 24H00489, 21H00273, 23H02101, 26H01674, and 26K01641), Japan Science, and Technology Agency (JPMJCR25T3), and Mitsubishi Foundation.

## Conflicts of Interest

The authors declare no conflicts of interest.

## Supporting information

Supplementary Material

Supplementary Material

Supplementary Material

Supplementary Material

## Data Availability

The data supporting the findings of this study are available from the corresponding authors upon reasonable request.
