# Supplementary material for: Development of a Photostable pH Biosensor Based on mStayGold
Source: Chembiochem. 2026 Aug 26;27(16):e70512. doi: 10.1002/cbic.70512 (PMC13509110; doi:10.1002/cbic.70512)
Supplement: Supplementary file 1 [file CBIC-27-e70512-s002.pdf]

Supplementary materials

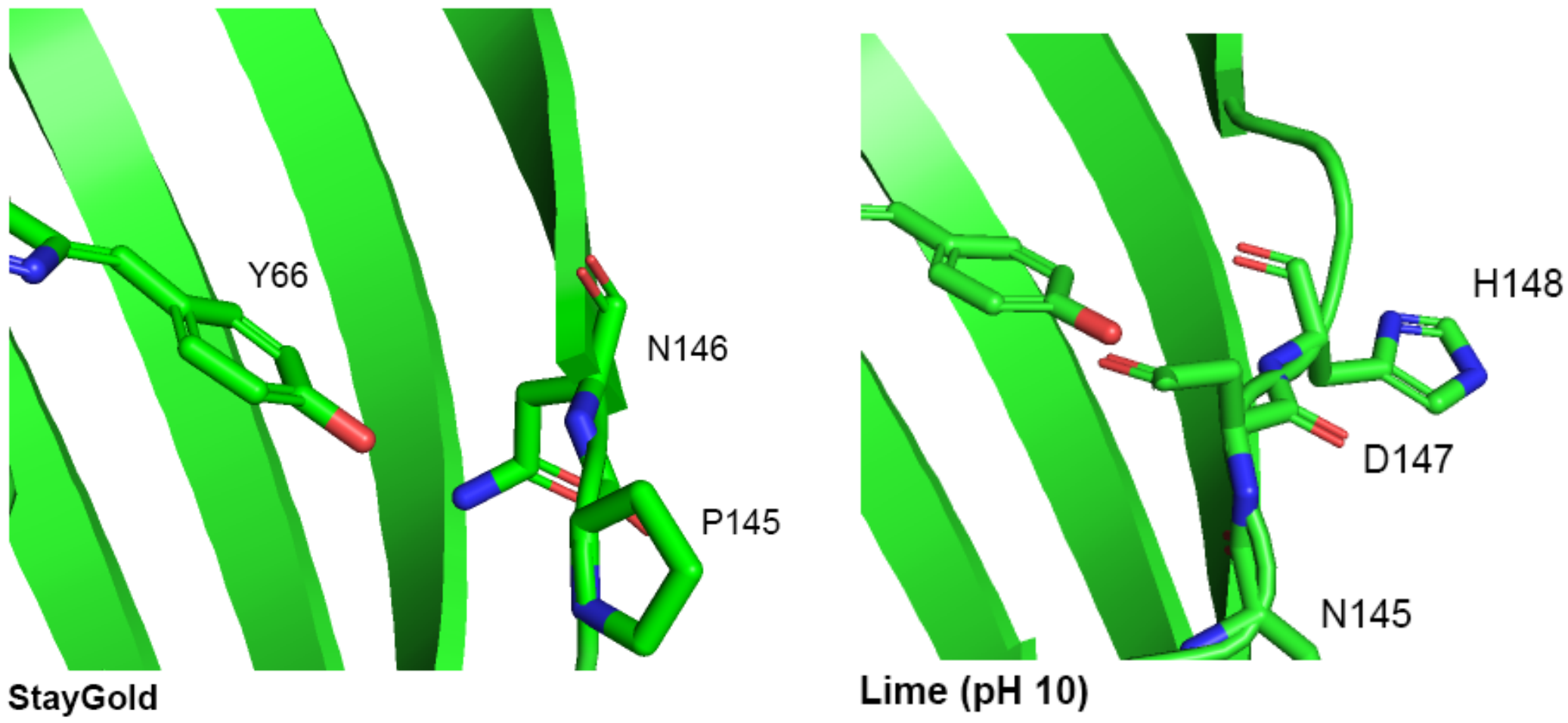

Supplementary Figure 1. Crystal structure comparison of the chromophore environments of StayGold (PDB ID: 8BXT, left) and Lime at pH 10 (PDB ID: 7YV3, right). The mutation D147 in Lime, whose side chain points inside the β-barrel, is thought to be a major cause of its elevated pK_a. We hypothesized that mutations to the analogous residues in StayGold, particularly into polar residues with negatively charged side chains, would produce a similar result.

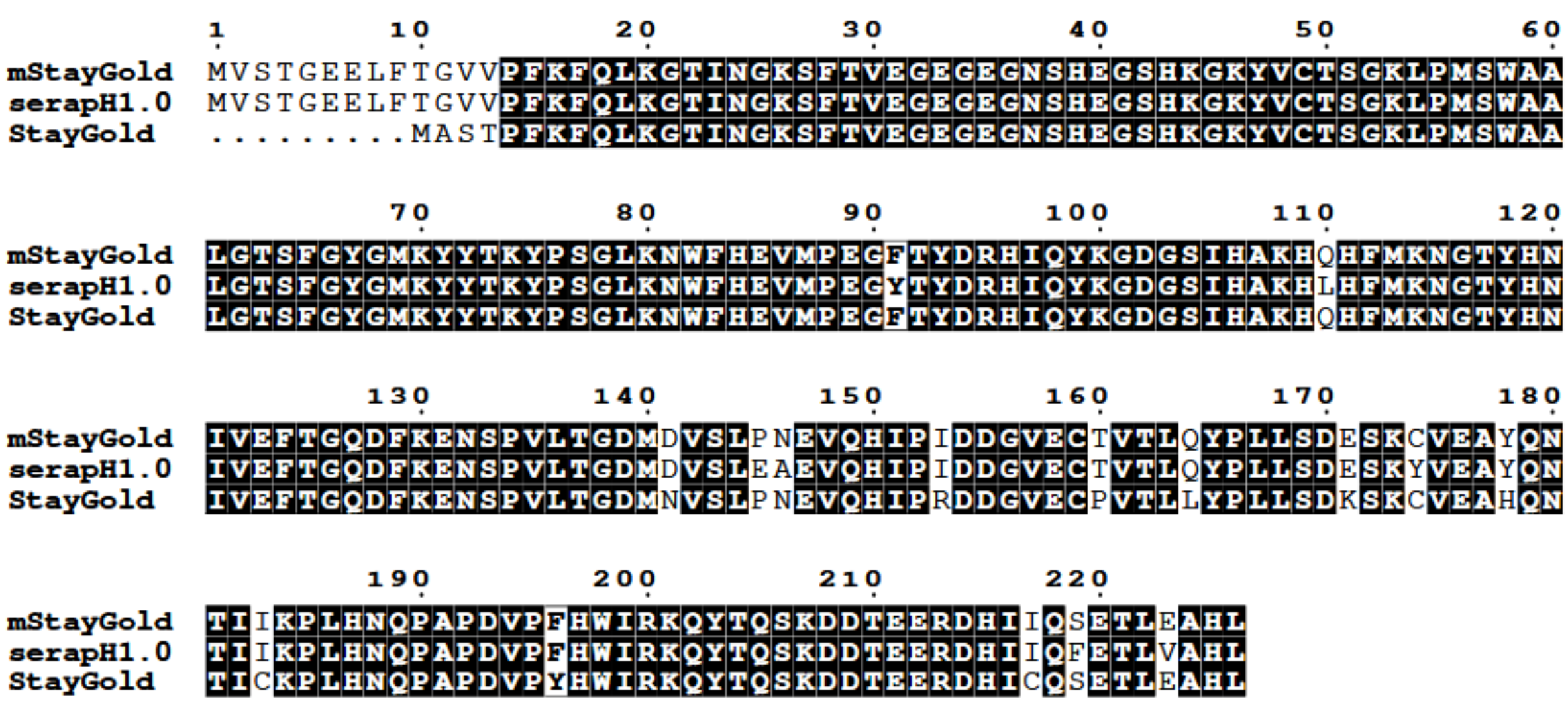

Supplementary Figure 2. Sequence alignment of StayGold, mStayGold(J), and serapH1.0. Alignment visualization was performed with ESPript [1].

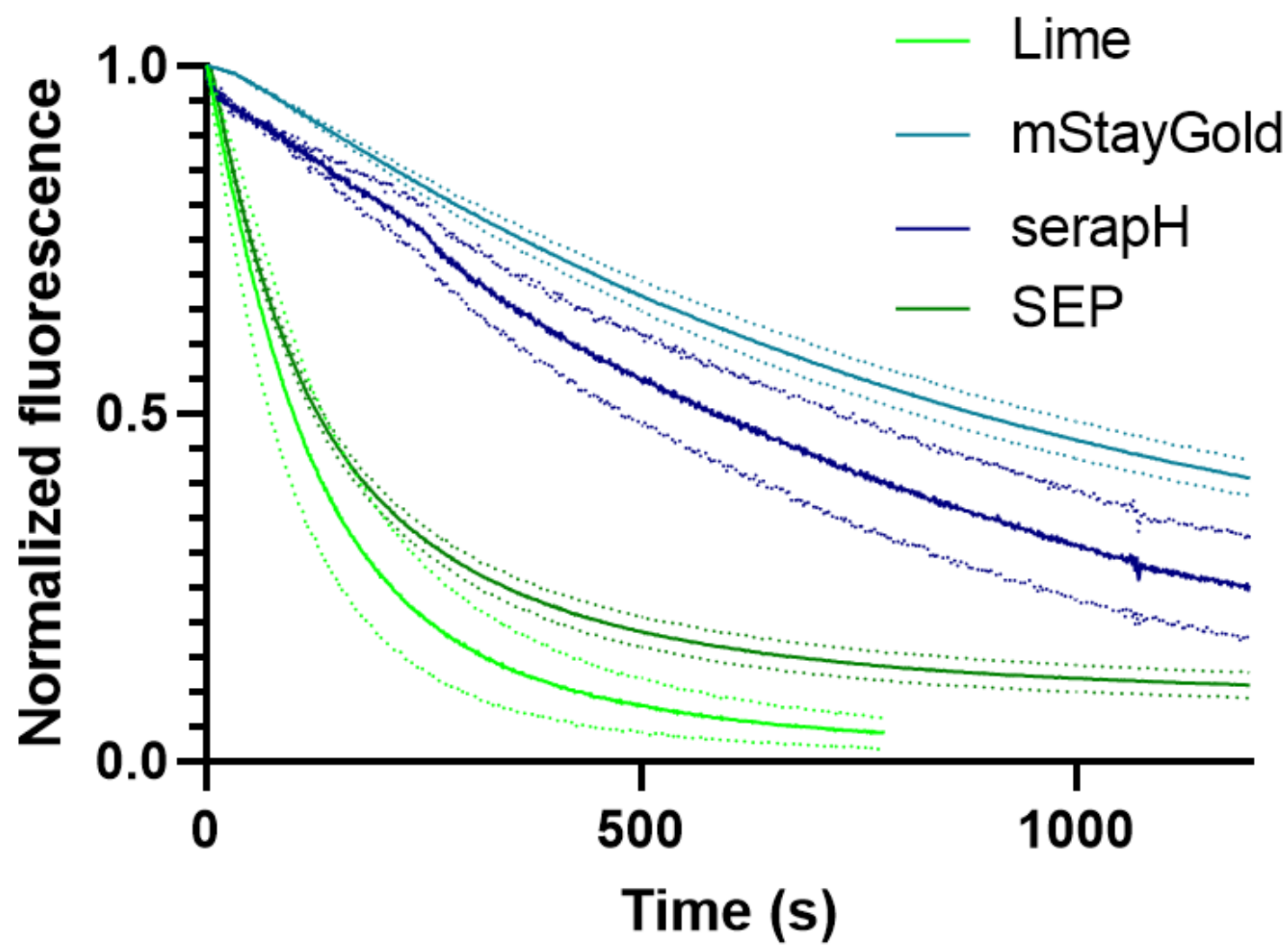

Supplementary Figure 3. Photostability measurements of serapH and controls at pH 5.5. To mitigate quenching effects in pH-sensitive proteins, Lime, serapH, and SEP were assayed at 50 µM, and mSG at 10 µM concentration.

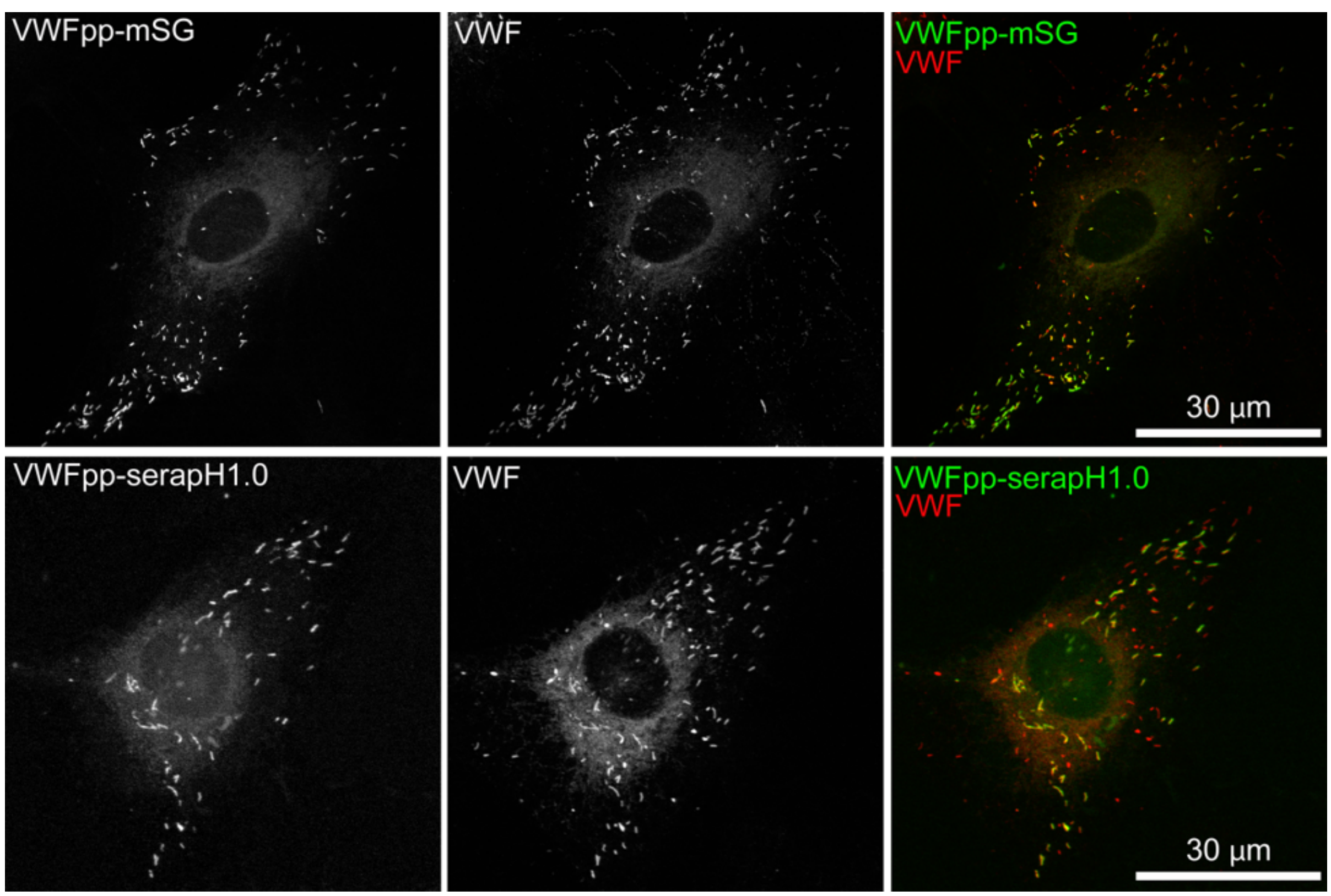

Supplementary Figure 4. VWFpp-serapH1.0 is correctly trafficked to Weibel-Palade bodies in HUVEC. Representative confocal fluorescence images of cultured HUVEC 24 hours post-transfection with VWFpp-mSG (Top left panel,

green in colour merge) or VWFpp-serapH1.0 (bottom left panel, green in colour merge image) and immunolabelled for endogenous VWF to reveal WPBs (middle panels, red in colour merge image).

Supplementary Table 1. Commonly used pH-sensitive FPs and their spectral characteristics. N.D. denotes values that are not reported in literature.

FP	Parent FP	Excitation maxima (nm)	Emission maxima (nm)	Extinction coefficient (EC, M^{-1} cm^{-1})	Quantum yield (QY)	Mol. brightness (EC*QY/1000, mM^{-1} cm^{-1})	pK_a	$\Delta F/F_0$ (pH 5.5 to 7.4)
EGFP[2]	avGFP	488	509	57,000	0.60	34	5.5	N.D.
SEP[2]	EGFP	488	511	37,000	0.80	30	7.0	66
Ratiomet. pHluorin[3]	avGFP	475	509	N.D.	N.D.	N.D.	7.1	N.D.
E^2GFP[4]	EGFP	508	523	29,000	0.91	26	6.8	N.D.
Lime[2]	sfGFP	488	509	45,000	0.77	35	7.1	82
deGFP4[5]	avGFP	509	518	27,000	0.15	4.1	7.4	N.D.
serapH	mStay Gold(J)	506	516	110,000	0.94	100	7.1	21

Supporting Videos.

Videos S1-3. Examples of histamine-evoked exocytosis of VWFpp-serapH1.0 containing Weibel-Palade bodies in HUVEC.

Video clips (avi format, jpg compression in ImageJ (http://rsb.info.nih.gov/ij/)) of live HUVEC expressing VWFpp-serapH1.0 (24 hours post transfection). Videos show histamine-triggered exocytosis of Weibel-Palade bodies as discreet abrupt flashes of fluorescence arising from the pH-dependent change in VWFpp-serapH1.0 fluorescence. Near the end of each video addition of 10 mM NH_4Cl reveals non-exocytosed WPBs. Movies are 5 frame averages of the raw data (acquired at 10 frames per second). Time elapsed from the start of the video clip is shown top right. Scale bars are 20 micrometers.

Supplementary references

1. Gouet, P., Robert, X. & Courcelle, E. ESPript/ENDscript: Extracting and rendering sequence and 3D information from atomic structures of proteins. *Nucleic Acids Res.* **31**, 3320–3323 (2003).
2. Shen, Y. *et al.* Rational engineering of an improved genetically encoded pH sensor based on superecliptic pHluorin. *ACS Sens.* **8**, 3014–3022 (2023).
3. Miesenböck, G., De Angelis, D. A. & Rothman, J. E. Visualizing secretion and synaptic transmission with pH-sensitive green fluorescent proteins. *Nature* **394**, 192–195 (1998).
4. Bizzarri, R. *et al.* Development of a novel GFP-based ratiometric excitation and emission pH indicator for intracellular studies. *Biophys. J.* **90**, 3300–3314 (2006).
5. Hanson, G. T. *et al.* Green fluorescent protein variants as ratiometric dual emission pH sensors. 1. Structural characterization and preliminary application. *Biochemistry* **41**, 15477–15488 (2002).
